# Changing associations of coronary heart disease incidence with current partnership status and marital history over three decades

**DOI:** 10.1016/j.ssmph.2022.101080

**Published:** 2022-03-26

**Authors:** Karri Silventoinen, Kaarina Korhonen, Pekka Martikainen

**Affiliations:** a University of Helsinki, Faculty of Social Sciences, Population Research Unit, Finland; b University of Helsinki, Faculty of Medicine, Department of Public Health, Finland; c Stockholm University, Department of Public Health Sciences, Sweden; d Max-Planck-Institute for Demographic Research, Germany

**Keywords:** Marital status, CHD incidence, Temporal change, Partnership history

## Abstract

Married men and women have better health than non-married, but little is known about how cohabitation and marital history are associated with coronary heart disease (CHD) incidence and how these associations have changed over time. We analyzed these associations by fitting Cox regression models to register data covering the whole Finnish population aged 35 years or older (N = 4,415,590), who experienced 530,560 first time non-fatal or fatal CHD events during the years 1990–2018. Further, we used stratified Cox regression models to analyze CHD incidence within same-sex sibling pairs (N = 377,730 pairs). Married men and women without previous divorce had the lowest CHD incidence whereas cohabitation and a history of divorce were associated with higher CHD incidence. The associations were stronger in younger (35–64 years old) than older participants (65 years or older). These associations remained after adjusting for several indicators of social position, and the lower CHD incidence among those married without previous divorce was also observed within sibling pairs with a shared family background. The differences in CHD incidence between the categories generally widened over time; the largest and most systematic widening was observed among women in the younger age category. The long standing negative effect of divorce suggests that selection may partly explain the association between partnership status and CHD incidence. Partnership status is an increasingly important factor contributing to social inequalities in health.

## Highlights

• Married men and women without previous divorce history have the lowest CHD risk.
• Cohabitation is associated with higher CHD risk than marriage.
• The associations between partnership and CHD risk are not explained by social factors.
• These associations generally widened over time, especially among women.

Marital status has received generally less attention in social epidemiology than some other social indicators, such as education and income. However, the importance of marital status as a social determinant of health has increased in recent decades when mortality differences between married and non-married persons have widened (Roelfs et al., 2011). This development has also affected gender differences in the association between marital status and health; when in the earlier studies the mortality difference between married and non-married persons was larger in men as compared to women, in the most recent studies this difference was roughly similar in men and women (Roelfs et al., 2011). Most of the studies concerning the association between marriage and health have concerned Western countries and different-sex marriages, but health benefits of marriage have also been found in other cultural contexts (Jee & Cho, 2019) and for same-sex marriages (Solazzo et al., 2020) demonstrating the universality of this association. However, much less is known about the associations between marital status and specific diseases. In this context, coronary heart disease (CHD) is especially important since it is not only the leading cause of death both globally and in Western societies (Lozano et al., 2012), but it is also strongly socially patterned (Mackenbach et al., 2000). In Northern European countries, CHD is the most important cause of socio-economic mortality inequalities (Kulhánová et al., 2014). Thus, understanding the determinants of CHD mortality is essential for both enhancing public health in general and for reducing social health inequalities.

Previous studies have shown that CHD incidence is higher in non-married as compared to married men and women (Wong et al., 2018). However, not only having never married, but also marital dissolution due to divorce (Shor, Roelfs, Bugyi, & Schwartz, 2012) or spousal death (Shor, Roelfs, Curreli, et al., 2012), is associated with higher mortality, CHD in particular (Martikainen & Valkonen, 1996). There is also previous evidence that in addition to current marital status, also marital history affects mortality (Blomgren et al., 2012; Grundy & Tomassini, 2010). The differences in CHD risk according to marital status can result from both selection to marriage (or divorce in those currently married) according to health and health related factors and beneficial effects of marriage on health directly or through health behavior (Umberson & Thomeer, 2020). The health effects of marriage can also vary according to marital quality; marital quality may decline over time (Umberson et al., 2006) and women typically report more marital stress than men (Umberson & Williams, 2005), which can also affect physical health. Further, socio-economic factors can partly explain these associations since especially in men low social position is associated with lower probability of marriage (Jalovaara, 2012) and higher risk of divorce (Jalovaara, 2013). These differences may also stem from childhood. Low social position of childhood family is associated with both higher CHD risk in adulthood (Kilpi et al., 2017) and higher likelihood to experience parental divorce (Jalovaara & Andersson, 2018), which can further affect later union formation and dissolution patterns (Amato & DeBoer, 2001). Thus, when studying the association between marital status and health, it is important to take into account both childhood family background and marital history.

During the last decades, there has been a dramatic change in the formation of partnerships in Europe and the USA. Marriage has become a less common form of partnership and the rates of co-habitation and divorce have increased (Perelli-Harris et al., 2017). However, previous studies analyzing temporal changes in the association between partnership status and health have mainly focused on legal marriages and thus cannot capture the complexity of the changing forms of partnership. In this study, we aim to shed new light on the changing dynamics between partnership status, marital history and CHD incidence in the Finnish population over the last three decades. Based on detailed longitudinal register-based information, we can identify both cohabitation and divorce histories, and thus contribute to better understanding of how partnership characteristics are associated with CHD risk over time. Further, we use a quasi-experimental design of same-sex sibling pairs differing in their marital status. From the life course perspective, childhood family environment can importantly contribute to the association between marital status and health (Umberson & Thomeer, 2020), but measuring it retrospectively is difficult. Comparing discordant sibling pairs offers a powerful method to adjust for unobserved confounders shared by siblings and provide stronger evidence on causality. Finland provides an intriguing setting to study the changing dynamics between partnership history and health since, along with other Nordic countries, Finland has been a forerunner both in co-habitation and divorce followed by other European countries and the USA (Thomson, 2014).

## 1 Data and methods

We used data covering the whole Finnish population aged 35 years or older and residing in Finland in any year between 1990 and 2018. The population data from Statistics Finland were linked to administrative health registers using personal identification codes. The information from non-fatal CHD events were based on Hospital Discharge Register (ICD-10 codes I20.0 and I21–I22) and the fatal events on National Mortality Register (ICD-10 codes I20–I25, I46, R96 and R98). Because of the universal healthcare system in Finland, the hospital discharge register covers the whole Finnish population and includes virtually all non-fatal CHD events needing hospital-level care (Pajunen et al., 2005). Based on the Finnish law, also the mortality register covers the whole Finnish population. During the whole follow-up period from 1990 to 2018, we observed 530,560 incident CHD events (224,125 first time hospitalizations because of CHD and 306,435 CHD deaths without a prior hospitalization because of CHD) over 76,112,417 person-years at risk.

Current partnership status and marital history, abbreviated as marital history in the further sections, was classified as married, cohabiting, divorced, never married and widowed. Married and cohabiting participants were further stratified to those previously divorced and never divorced, and currently divorced to those divorced within the previous three years and those divorced more than three years ago. For those who had married after divorce and then re-divorced, the time to divorce was calculated from the latest divorce. Since the number of widowed who later married or cohabited was small (<1% of the study participants), no separate categories were used and they were classified as married or cohabiting. Information on marriage, divorce and widowhood was derived from the population register starting from 1987 and population censuses conducted in 1970, 1975, 1980, and 1985. Cohabitation was based on linkage between the population and household registers and defined as two non-married persons of different genders living together, not close relatives and with an age difference less than 16 years. Thus, same-gender cohabiting partners were classified as singles.

Other covariates were having any children younger than 18 years living in the household (a binary variable), education (basic education only, secondary education and tertiary education), economic activity (employed, unemployed, student, pensioner and others), social class (upper non-manual, lower non-manual with independent work or subordinates, lower non-manual with routine work, specialized manual, non-specialized manual, self-employed farmer, entrepreneur and no known occupation) and personal incomes. Previous studies have shown that socioeconomic resources are associated with union formation (Jalovaara, 2012), union dissolution (Jalovaara, 2013) and CHD risk (Mackenbach et al., 2000). Thus, adjusting for these covariates gives more information on how possible compositional changes in socioeconomic characteristics can explain temporal changes in the associations between marital history and CHD risk. Information on all covariates was derived from the population register except for personal incomes, which were derived from the Tax Administration database including all annual taxable incomes and social benefits.

We estimated hazard ratios (HR) according to marital history in Cox regression models for first incident CHD event (non-fatal or fatal case without a prior hospitalization) with 95% confidence intervals (CI). The follow-up period was divided into 5-year periods with baselines at 1990, 1995, 2000, 2005, 2010 and 2015 to analyze how the associations between marital history and incident CHD events had changed over the three decades. Information on marital history and covariates were based on the situation at the time of each separate baseline, and the HRs were calculated for incident CHD events before the next baseline (5-year follow-up periods except the latest period with a 3-year follow-up) to minimize the dilution effect due to changes in partnership status during the follow-up. All individuals with a CHD hospitalization before each baseline were removed from the data. Those who died from other causes than CHD were censored at the time of death. Since previous studies have found larger mortality differentials in young and middle aged adults compared to older adults (Roelfs et al., 2011), we stratified the analyses by two age-groups: those aged 35–64 years and those aged 65 years or more. We also adjusted for attained age in years within the age-group strata in all models. Cox proportional hazards assumptions were not violated when tested graphically (Kaplan-Meier curves available from the corresponding author). Additionally, we calculated population attributable fractions (PAF) to evaluate the population level importance of marital history and to account for the changing distribution of marital history categories across the decades. PAF indicates the proportion of CHD cases that would have been avoided if the whole population had the same CHD risk as those in the lowest risk category.

We continued the analyses by studying the association between marital status and incident CHD events within sibling pairs. This method allows adjusting for unobserved childhood confounders since siblings share not only their childhood family but also many other environmental factors related to school and neighborhood. We selected one same-sex sibling pair from each family favoring the smallest age difference between siblings to minimize changes in family environment. The maximum age difference between siblings was limited to 5 years and only full biological siblings were selected. Together, we observed 195,787 brother and 181,943 sister pairs in the data. Since the selection of sibling pairs needed linkage between children and parents and this information was available only for the youngest birth cohorts (those born in 1950 or later), the median age in these analyses was somewhat younger (46 years) than in the population based analyses of 35–64-year-olds (49 years), and the earliest included baseline year was 1995. Since there was less statistical power in these analyses as compared to the individual level analyses, we dichotomized the variable as those married without previous divorce (the reference category) and others. The analyses were performed using stratified Cox regression models allowing a separate baseline hazard for each sibling pair. If an association between marital status and CHD risk is found within sibling pairs, a causal interpretation of the association between marital status and CHD risk gains credibility. To compare these within-pair estimates to population based estimates, we repeated the population based analyses within the cohort used in the sibling pair analyses, correcting the standard errors for clustering in sibling pairs. All statistical models were estimated by Stata statistical package, version 16.0. (College Station, TX: StataCorp LP).

## 2 Results

Table 1 presents the population distribution and CHD incidence by categories of marital history over the total follow-up period. The proportion of those who were married and had not previously divorced steadily declined from the year 1990–2015 in men and in 35–64-year-old women. This decline was explained by the increasing proportions of those who were divorced, cohabiting and never-married. In women 65 years of age or older, the proportion of married without previous divorce increased, which was because of decreasing proportion of widowed. The decreasing proportion of widowed was also seen in men 65 years of age or older and 35–64-year-old women.

**Table 1:** Proportions of population and incidence rates of coronary heart disease events by marital history, baseline year, age and gender.

Marital history	% of total baseline population						CHD incidence per 100,000 person years					
	1990	1995	2000	2005	2010	2015	1990	1995	2000	2005	2010	2015
**Men 35–64 years of age**												
Married	68	62	57	53	51	48	464	435	423	365	347	299
Married (divorced)	3	4	5	6	7	7	402	467	482	429	447	415
Cohabiting	4	5	7	8	9	10	300	258	266	247	277	249
Cohabiting (divorced)	3	4	5	5	5	5	454	504	535	470	430	409
Divorced (<3 years)	2	2	2	2	2	2	422	418	413	404	374	321
Divorced (≥3 years)	5	7	8	8	8	8	758	753	796	751	736	613
Never married	14	15	16	17	18	19	544	483	469	444	421	371
Widowed	1	1	1	1	1	1	1198	1066	980	917	737	677
N	952714	1008206	1058064	1077272	1065054	1033195	22691	22838	23793	21743	20769	10688
**Men 65 years of age or older**												
Married	70	69	68	66	64	60	2773	2633	2494	2021	1718	1443
Married (divorced)	1	1	2	3	4	6	2656	2333	2000	1644	1436	1194
Cohabiting	1	1	1	1	2	2	3186	3093	2765	2392	1711	1407
Cohabiting (divorced)	1	1	1	2	3	4	2686	2690	2326	1895	1470	1366
Divorced (<3 years)	0	0	0	0	0	0	2730	2636	2156	2009	1801	1451
Divorced (≥3 years)	4	5	6	7	9	10	3157	3074	3143	2694	2181	1902
Never married	8	8	9	9	8	9	3218	3131	3058	2835	2424	1995
Widowed	16	14	13	11	10	9	4198	4189	4471	3985	3534	3134
N	214554	240025	260031	287346	336217	423835	26973	29221	30907	28963	28813	19561
**Women 35–64 years of age**												
Married	66	63	58	54	52	50	118	122	134	119	115	97
Married (divorced)	3	4	4	6	7	7	85	114	166	150	150	153
Cohabiting	3	4	6	7	8	9	99	87	82	70	85	75
Cohabiting (divorced)	3	4	4	5	5	5	113	138	157	154	156	146
Divorced (<3 years)	2	2	2	2	2	2	87	75	101	91	99	77
Divorced (≥3 years)	8	9	11	11	12	11	183	208	215	203	210	191
Never married	10	10	11	12	12	14	140	130	139	112	129	123
Widowed	6	5	4	3	3	2	349	361	354	294	291	281
N	972581	1017975	1067198	1090360	1082559	1044504	6565	7035	7918	7157	7208	3723
**Women 65 years of age or older**												
Married	31	33	35	37	39	41	1382	1343	1279	1026	868	688
Married (divorced)	0	0	1	1	2	3	1145	1288	1032	897	713	569
Cohabiting	1	1	1	1	1	2	1653	1888	1798	1211	1004	839
Cohabiting (divorced)	0	0	1	1	1	2	1433	1401	1167	953	744	685
Divorced (<3 years)	0	0	0	0	0	0	1226	1308	1272	773	645	834
Divorced (≥3 years)	6	7	9	10	12	14	2023	1884	1903	1486	1269	1024
Never married	13	11	10	9	9	8	2017	2112	2255	1929	1627	1255
Widowed	49	46	44	40	35	30	2479	2647	2851	2585	2309	1978
N	410141	434914	446106	463842	506199	592714	36115	39071	40854	35948	32826	19180

We first studied how marital history was associated with CHD risk in 35–64-year-olds. In men, the CHD risk was lowest among those married without previous divorce history and highest among those who had been divorced for 3 or more years (Table 2). The differences increased from the first five-year follow-up period starting in 1990 (PAF = 0.10) until the period starting in 2005 (PAF = 0.18), but after that remained unchanged. Adjusting for the indicators of socioeconomic position explained part of these differences: the PAFs decreased by 20–50% (Model 2). In women, the lowest risk was among married without previous divorce and the highest risk among the cohabiting who had previously divorced or those who had been divorced for 3 or more years depending on the follow-up period (Table 3). The differences increased over time: the PAF was 0.08 in the first follow-up period, and it increased up to 0.18 in the last follow-up period starting in 2015. The indicators of socioeconomic position explained a slightly smaller proportion of these differences (17–28%) than in men (Model 2). Women had smaller differences in CHD risk between the marital history categories in all other periods (p-value for gender interaction <0.0001) than the latest period when the PAF was slightly greater than in men (p-value for gender interaction 0.123).

**Table 2:** Hazard rations (HR) of coronary heart disease events by marital history in men of 35–64 years of age at baselines.

Marital history	Baseline year																	
	1990			1995			2000			2005			2010			2015		
	HR	95% CI		HR	95% CI		HR	95% CI		HR	95% CI		HR	95% CI		HR	95% CI	
		LL	UL		LL	UL		LL	UL		LL	UL		LL	UL		LL	UL
**Model 1**																		
Married	1.00			1.00			1.00			1.00			1.00			1.00		
Married (divorced)	1.10	1.01	1.20	1.18	1.10	1.27	1.16	1.09	1.23	1.14	1.08	1.21	1.20	1.14	1.26	1.18	1.10	1.27
Cohabiting	1.30	1.19	1.42	1.22	1.13	1.32	1.26	1.18	1.35	1.23	1.16	1.32	1.27	1.20	1.34	1.21	1.12	1.31
Cohabiting (divorced)	1.28	1.19	1.38	1.35	1.27	1.44	1.39	1.31	1.47	1.33	1.25	1.41	1.20	1.12	1.27	1.26	1.15	1.37
Divorced (<3 years)	1.34	1.21	1.49	1.43	1.30	1.58	1.39	1.27	1.53	1.52	1.38	1.68	1.44	1.30	1.60	1.40	1.22	1.61
Divorced (≥3 years)	1.53	1.46	1.61	1.64	1.57	1.72	1.74	1.67	1.81	1.81	1.74	1.88	1.76	1.69	1.84	1.61	1.51	1.70
Never married	1.44	1.39	1.50	1.49	1.43	1.55	1.54	1.49	1.60	1.65	1.59	1.71	1.51	1.46	1.57	1.44	1.37	1.52
Widowed	1.34	1.23	1.46	1.40	1.28	1.53	1.44	1.30	1.59	1.62	1.46	1.80	1.35	1.20	1.53	1.38	1.15	1.67
PAF	0.10	0.09	0.11	0.13	0.12	0.14	0.15	0.14	0.16	0.18	0.17	0.19	0.17	0.16	0.18	0.16	0.14	0.18
**Model 2**																		
Married	1.00			1.00			1.00			1.00			1.00			1.00		
Married (divorced)	1.11	1.02	1.21	1.18	1.10	1.26	1.16	1.09	1.23	1.13	1.07	1.20	1.18	1.11	1.24	1.15	1.07	1.24
Cohabiting	1.13	1.03	1.24	1.07	0.99	1.16	1.12	1.05	1.20	1.10	1.03	1.17	1.14	1.07	1.20	1.09	1.01	1.18
Cohabiting (divorced)	1.17	1.09	1.26	1.23	1.16	1.31	1.28	1.21	1.36	1.23	1.15	1.30	1.11	1.04	1.18	1.17	1.07	1.27
Divorced (<3 years)	1.24	1.12	1.38	1.30	1.18	1.44	1.29	1.17	1.42	1.39	1.26	1.54	1.33	1.20	1.47	1.30	1.13	1.49
Divorced (≥3 years)	1.31	1.24	1.37	1.39	1.33	1.45	1.48	1.42	1.54	1.51	1.45	1.57	1.50	1.44	1.56	1.37	1.29	1.46
Never married	1.18	1.13	1.22	1.18	1.14	1.23	1.22	1.17	1.27	1.26	1.20	1.31	1.19	1.14	1.24	1.15	1.08	1.21
Widowed	1.24	1.14	1.35	1.29	1.18	1.42	1.31	1.19	1.45	1.47	1.33	1.64	1.23	1.09	1.39	1.25	1.04	1.51
PAF	0.08	0.07	0.09	0.10	0.09	0.11	0.12	0.10	0.13	0.11	0.09	0.12	0.10	0.07	0.12	0.08	0.07	0.09

Model 1: Adjusted for age at the baseline.

Model 2: Adjusted for age at the baseline, having any children under 18 years of age in the family, education, occupation based socio-economic position, employment status and personal incomes.

**Table 3:** Hazard rations (HR) of coronary heart disease events by marital history in women of 35–64 years of age at baselines.

Marital history	Baseline year																	
	1990			1995			2000			2005			2010			2015		
	HR	95% CI		HR	95% CI		HR	95% CI		HR	95% CI		HR	95% CI		HR	95% CI	
		LL	UL		LL	UL		LL	UL		LL	UL		LL	UL		LL	UL
**Model 1**																		
Married	1.00			1.00			1.00			1.00			1.00			1.00		
Married (divorced)	1.22	1.01	1.47	1.25	1.08	1.44	1.45	1.31	1.61	1.36	1.24	1.50	1.31	1.20	1.44	1.44	1.28	1.62
Cohabiting	1.41	1.19	1.67	1.36	1.17	1.58	1.23	1.08	1.40	1.13	1.00	1.28	1.26	1.13	1.40	1.17	1.01	1.35
Cohabiting (divorced)	1.56	1.34	1.83	1.54	1.36	1.74	1.44	1.29	1.60	1.44	1.30	1.60	1.36	1.23	1.51	1.40	1.22	1.61
Divorced (<3 years)	1.35	1.08	1.69	1.11	0.88	1.39	1.27	1.06	1.54	1.23	1.01	1.51	1.36	1.12	1.65	1.20	0.91	1.59
Divorced (≥3 years)	1.31	1.21	1.42	1.49	1.39	1.60	1.40	1.31	1.50	1.45	1.36	1.55	1.49	1.39	1.59	1.51	1.38	1.66
Never married	1.13	1.04	1.23	1.18	1.09	1.29	1.29	1.20	1.39	1.21	1.11	1.31	1.38	1.28	1.48	1.43	1.29	1.58
Widowed	1.22	1.14	1.31	1.38	1.28	1.49	1.39	1.29	1.51	1.41	1.29	1.55	1.47	1.32	1.62	1.62	1.39	1.89
PAF	0.08	0.06	0.10	0.12	0.10	0.14	0.13	0.11	0.15	0.13	0.11	0.15	0.16	0.14	0.18	0.18	0.15	0.21
**Model 2**																		
Married	1.00			1.00			1.00			1.00			1.00			1.00		
Married (divorced)	1.19	0.99	1.44	1.19	1.03	1.37	1.40	1.26	1.55	1.30	1.18	1.44	1.26	1.15	1.38	1.36	1.21	1.53
Cohabiting	1.29	1.09	1.53	1.22	1.05	1.42	1.11	0.98	1.27	1.06	0.93	1.20	1.18	1.05	1.32	1.08	0.93	1.25
Cohabiting (divorced)	1.42	1.22	1.67	1.38	1.22	1.56	1.31	1.17	1.45	1.32	1.19	1.47	1.27	1.15	1.41	1.30	1.13	1.49
Divorced (<3 years)	1.36	1.09	1.70	1.09	0.87	1.36	1.25	1.03	1.51	1.21	0.99	1.47	1.31	1.08	1.58	1.14	0.86	1.50
Divorced (≥3 years)	1.29	1.19	1.40	1.40	1.31	1.51	1.29	1.21	1.38	1.34	1.25	1.43	1.37	1.28	1.46	1.35	1.23	1.48
Never married	1.10	1.01	1.20	1.07	0.98	1.17	1.12	1.04	1.21	1.03	0.95	1.12	1.18	1.09	1.28	1.18	1.06	1.31
Widowed	1.26	1.17	1.36	1.36	1.25	1.47	1.30	1.19	1.41	1.37	1.24	1.51	1.38	1.25	1.53	1.50	1.28	1.74
PAF	0.08	0.06	0.10	0.10	0.08	0.12	0.10	0.08	0.12	0.10	0.08	0.12	0.12	0.10	0.15	0.13	0.09	0.16

Model 1: Adjusted for age at the baseline.

Model 2: Adjusted for age at the baseline, having any children under 18 years of age in the family, education, occupation based socio-economic position, employment status and personal incomes.

Table 4 presents the corresponding analyses for men 65 years of age or older. Also in this age category, married had generally the lowest risk of CHD, but the differences in CHD risk between the marital history categories were narrower as compared to the 35–64 years category (reduction in PAFs 25–55%). The adjustment for socio-economic indicators slightly decreased the PAFs (10–20%, Model 2). In women, married had generally the lowest risk and the differences were narrower than in the younger age category (reduction in PAFs by 12–42% except during the follow-up period starting in 2005 where no difference in PAFs was seen) (Table 5). The PAFs were higher in women than in men in this age category in all follow-up periods indicating larger differences in CHD risk between marital history categories in women (p-values for gender interaction <0.0001). Among women, the effect of the adjustment for socioeconomic indicators was opposite than in men, and it increased PAFs (7–33%) except in the follow-up period starting in 2000 where changes in PAFs were not observed (Model 2).

**Table 4:** Hazard rations (HR) of coronary heart disease events by marital history in men of 65 years of age or older at baselines.

Marital history	Baseline year																	
	1990			1995			2000			2005			2010			2015		
	HR	95% CI		HR	95% CI		HR	95% CI		HR	95% CI		HR	95% CI		HR	95% CI	
		LL	UL		LL	UL		LL	UL		LL	UL		LL	UL		LL	UL
**Model 1**																		
Married	1.00			1.00			1.00			1.00			1.00			1.00		
Married (divorced)	1.11	0.95	1.29	1.03	0.91	1.16	0.95	0.87	1.05	1.01	0.93	1.09	1.07	1.00	1.14	1.02	0.96	1.10
Cohabiting	1.12	1.00	1.25	1.17	1.05	1.30	1.12	1.01	1.24	1.24	1.13	1.37	1.09	0.99	1.20	1.14	1.03	1.27
Cohabiting (divorced)	1.09	0.94	1.26	1.21	1.07	1.35	1.14	1.03	1.26	1.19	1.09	1.30	1.08	1.00	1.17	1.18	1.08	1.28
Divorced (<3 years)	1.10	0.88	1.38	1.15	0.92	1.45	1.07	0.84	1.36	1.23	0.98	1.56	1.39	1.12	1.72	1.37	1.05	1.77
Divorced (≥3 years)	1.18	1.11	1.25	1.24	1.18	1.31	1.37	1.31	1.43	1.48	1.41	1.54	1.44	1.38	1.50	1.49	1.43	1.56
Never married	1.17	1.12	1.22	1.25	1.20	1.30	1.28	1.23	1.34	1.46	1.41	1.52	1.48	1.42	1.54	1.52	1.45	1.59
Widowed	1.15	1.11	1.19	1.13	1.10	1.17	1.20	1.16	1.24	1.26	1.22	1.30	1.26	1.22	1.31	1.27	1.22	1.33
PAF	0.05	0.04	0.05	0.05	0.05	0.06	0.07	0.06	0.08	0.10	0.09	0.11	0.10	0.09	0.11	0.12	0.10	0.13
**Model 2**																		
Married	1.00			1.00			1.00			1.00			1.00			1.00		
Married (divorced)	1.14	0.98	1.33	1.07	0.95	1.21	1.00	0.91	1.11	1.05	0.97	1.14	1.11	1.03	1.18	1.06	0.99	1.13
Cohabiting	1.07	0.96	1.20	1.12	1.01	1.25	1.07	0.96	1.18	1.17	1.06	1.29	1.03	0.93	1.13	1.08	0.97	1.21
Cohabiting (divorced)	1.05	0.90	1.21	1.17	1.04	1.31	1.11	1.01	1.23	1.15	1.05	1.26	1.05	0.97	1.14	1.15	1.05	1.25
Divorced (<3 years)	1.09	0.87	1.37	1.15	0.92	1.45	1.07	0.84	1.36	1.23	0.97	1.54	1.40	1.13	1.73	1.35	1.04	1.76
Divorced (≥3 years)	1.13	1.07	1.21	1.20	1.14	1.27	1.32	1.27	1.39	1.41	1.35	1.48	1.38	1.32	1.43	1.43	1.36	1.50
Never married	1.11	1.06	1.16	1.18	1.13	1.23	1.20	1.15	1.25	1.35	1.29	1.40	1.34	1.29	1.40	1.38	1.31	1.45
Widowed	1.13	1.10	1.17	1.11	1.08	1.15	1.17	1.14	1.21	1.23	1.19	1.27	1.23	1.19	1.28	1.25	1.19	1.30
PAF	0.04	0.03	0.05	0.04	0.04	0.05	0.06	0.05	0.07	0.09	0.08	0.10	0.09	0.08	0.10	0.10	0.09	0.11

Model 1: Adjusted for age at the baseline.

Model 2: Adjusted for age at the baseline, having any children under 18 years of age in the family, education, occupation based socio-economic position, employment status and personal incomes.

**Table 5:** Hazard rations (HR) of coronary heart disease events by marital history in women of 65 years of age or older at baselines.

Marital history	Baseline year																	
	1990			1995			2000			2005			2010			2015		
	HR	95% CI		HR	95% CI		HR	95% CI		HR	95% CI		HR	95% CI		HR	95% CI	
		LL	UL		LL	UL		LL	UL		LL	UL		LL	UL		LL	UL
**Model 1**																		
Married	1.00			1.00			1.00			1.00			1.00			1.00		
Married (divorced)	1.02	0.76	1.37	1.20	0.98	1.47	1.04	0.88	1.24	1.19	1.04	1.36	1.15	1.03	1.28	1.11	0.99	1.24
Cohabiting	1.11	0.97	1.26	1.30	1.16	1.45	1.34	1.20	1.49	1.17	1.04	1.32	1.17	1.04	1.31	1.28	1.12	1.46
Cohabiting (divorced)	1.18	0.93	1.48	1.24	1.03	1.49	1.17	1.00	1.37	1.26	1.10	1.45	1.16	1.02	1.31	1.31	1.16	1.49
Divorced (<3 years)	1.06	0.75	1.49	1.15	0.81	1.63	1.28	0.90	1.81	1.02	0.67	1.54	1.08	0.72	1.61	1.74	1.18	2.58
Divorced (≥3 years)	1.17	1.11	1.22	1.11	1.07	1.16	1.19	1.15	1.24	1.20	1.15	1.25	1.26	1.21	1.31	1.29	1.23	1.36
Never married	0.97	0.93	1.01	1.00	0.97	1.04	1.09	1.05	1.13	1.15	1.10	1.19	1.20	1.15	1.25	1.26	1.19	1.34
Widowed	1.11	1.08	1.14	1.11	1.08	1.14	1.15	1.12	1.18	1.20	1.17	1.24	1.23	1.19	1.27	1.29	1.24	1.34
PAF	0.06	0.04	0.08	0.07	0.05	0.09	0.10	0.08	0.12	0.13	0.11	0.14	0.14	0.12	0.16	0.16	0.14	0.18
**Model 2**																		
Married	1.00			1.00			1.00			1.00			1.00			1.00		
Married (divorced)	1.06	0.79	1.42	1.23	1.00	1.51	1.06	0.89	1.26	1.22	1.07	1.40	1.18	1.06	1.31	1.13	1.01	1.26
Cohabiting	1.11	0.97	1.26	1.29	1.15	1.44	1.31	1.17	1.46	1.17	1.04	1.32	1.16	1.03	1.31	1.27	1.11	1.46
Cohabiting (divorced)	1.18	0.93	1.49	1.22	1.02	1.47	1.15	0.98	1.35	1.25	1.08	1.43	1.14	1.01	1.29	1.29	1.14	1.47
Divorced (<3 years)	1.07	0.76	1.50	1.17	0.83	1.66	1.28	0.91	1.82	1.04	0.69	1.58	1.12	0.75	1.67	1.77	1.19	2.62
Divorced (≥3 years)	1.20	1.15	1.26	1.13	1.08	1.18	1.20	1.15	1.24	1.21	1.16	1.26	1.27	1.22	1.32	1.30	1.23	1.36
Never married	1.02	0.99	1.06	1.05	1.01	1.09	1.13	1.09	1.17	1.21	1.16	1.26	1.26	1.21	1.32	1.32	1.25	1.39
Widowed	1.13	1.10	1.16	1.13	1.10	1.16	1.14	1.11	1.17	1.23	1.19	1.26	1.24	1.20	1.28	1.29	1.24	1.35
PAF	0.08	0.06	0.10	0.08	0.06	0.10	0.10	0.08	0.12	0.14	0.12	0.16	0.15	0.13	0.16	0.17	0.15	0.19

Model 1: Adjusted for age at the baseline.

Model 2: Adjusted for age at the baseline, having any children under 18 years of age in the family, education, occupation based socio-economic position, employment status and personal incomes.

Finally, we examined the role of childhood family environment by studying the difference in CHD risk within same-sex sibling pairs where one sibling was married and had not previously divorced and the other sibling belonged to any of the other marital history categories (Table 6). The systematically higher CHD risk compared to those married without previous divorce was seen in all follow-up periods in the within-pair analyses. The adjustment for within-pair differences in the socioeconomic indicators somewhat attenuated the HRs in men and women (Model 2). The HRs in men were slightly but systematically lower in the within-pair than individual level analyses. However, in women, the differences were unsystematic and both lower and higher HRs were seen in the within-pair analyses as compared to the individual level analyses.

**Table 6:** Hazard rations (HR) of coronary heart disease events within and between same-sex sibling pairs of all other marital categories compared to those married without previous divorce history by baseline year and gender.

Baseline year	Men						Women					
	Model 1			Model 2			Model 1			Model 2		
	HR	95% CI		HR	95% CI		HR	95% CI		HR	95% CI	
		LL	UL		LL	UL		LL	UL		LL	UL
**Within- pair analyses**												
1995	1.53	1.25	1.87	1.32	1.04	1.68	2.08	1.21	3.59	1.60	0.81	3.14
2000	1.39	1.23	1.58	1.27	1.09	1.47	1.55	1.17	2.05	1.29	0.95	1.76
2005	1.30	1.17	1.44	1.12	1.00	1.26	1.20	0.98	1.45	1.00	0.81	1.24
2010	1.20	1.09	1.31	1.10	1.00	1.21	1.43	1.21	1.69	1.29	1.09	1.53
2015	1.29	1.16	1.43	1.17	1.04	1.31	1.28	1.07	1.53	1.19	0.99	1.43
**Between- pair analyses**												
1995	1.58	1.39	1.79	1.23	1.03	1.47	1.70	1.23	2.35	1.28	0.89	1.84
2000	1.48	1.36	1.62	1.16	1.05	1.29	1.51	1.27	1.81	1.17	0.97	1.42
2005	1.42	1.33	1.53	1.13	1.04	1.22	1.37	1.19	1.57	1.12	0.98	1.30
2010	1.34	1.27	1.42	1.13	1.06	1.21	1.43	1.28	1.59	1.23	1.10	1.37
2015	1.40	1.31	1.51	1.19	1.11	1.28	1.39	1.24	1.56	1.23	1.09	1.39

Model 1: Adjusted for age at the baseline.

Model 2: Adjusted for age at the baseline, having any children under 18 years of age in the family, education, occupation based socio-economic position, employment status and personal incomes.

## 3 Discussion

In this study covering the whole population of Finland in years 1990–2018, we found that not only current partnership status but also marital history affected the CHD risk in men and women. Previous studies have consistently shown that marriage is associated with lower (Wong et al., 2018) and divorce with higher CHD risk (Martikainen et al., 2005). Our results showed that previous divorce increases the CHD risk also in men and women who are currently married or cohabiting. Among currently divorced, the CHD risk was higher in those who had been divorced at least 3 years as compared to those who had been divorced less than three years. This suggests that CHD risk is higher among those not finding a new partner after the divorce. These results are consistent with the hypothesis that selection mechanisms may partly explain the association between CHD risk and divorce status, and there may be same factors affecting CHD risk and the probability to divorce and find a new partner after the divorce. Health behavioral factors are likely candidates for these common factors since being married is associated with better health behavior, such as non-smoking and regular physical activity (Manfredini et al., 2017), whereas heavy use of alcohol is associated with a higher divorce risk (Collins et al., 2007). In a Scottish study, physical activity, smoking and alcohol use explained a third of the excess risk of CHD of divorced men and around fifth of divorced women when compared to married men and women (Molloy et al., 2009). Another explanation for these findings is that divorce has a long-standing effect on health not removed even by a new partnership. Both explanations received some support from a previous Finnish study on psychotropic medication use. The authors found higher levels of medication use among divorced than among continuously married already several years before the divorce, which peaked at the time of divorce and remained at a higher level over an eight-year period after the divorce (Metsä-Simola & Martikainen, 2013).

We also found that cohabiting men and women had higher CHD risk than those who were married. There are previous studies showing that heavy alcohol use (Joutsenniemi et al., 2007) and mental health problems (van Hedel et al., 2018) are more common in cohabiting than in married couples. Well-being is also found to be lower in cohabitants than married couples even in many European countries where cohabitation is a culturally widely accepted form of partnership (Soons & Kalmijn, 2009). This excess risk among cohabitants can thus be caused both by health behavior but also weaker emotional and task support from the cohabiting relationship. It is noteworthy that cohabitation usually leads to separation or marriage, with only a small minority staying in the cohabiting relationship for an extended period (Jalovaara & Hull, 2018). Thus, the cohabiting couples are a mixture of those who will eventually marry and those who will become non-married.

When studying these associations over time, we found that the differences increased more in middle aged women than in middle aged men leading to the disappearance of the gender difference in the association between marital history and CHD incidence in the latest follow-up period. A similar pattern of a narrowing gender gap in all-cause mortality between married and non-married has previously been reported in a meta-analysis (Roelfs et al., 2011), and thus this seems to be a universal trend. In the more recent follow-up periods, the differences between marital history categories were more strongly attributable to socioeconomic factors than in the earlier follow-up periods. During this study period in Finland, partnership status was an important factor behind income inequality in the middle-aged population (Erola & Kilpi-Jakonen, 2021). Our results indicate that socioeconomic inequalities between the marital history categories have increased during the follow-up period, also having implications for CHD risk. However, clear differences were also found after adjusting the results for adult socioeconomic position and when we adjusted the results for childhood family background by using a quasi-experimental design of discordant sibling pairs.

The differences in CHD risk between the marital history categories were substantially smaller in older participants as compared to middle-aged adults. This age difference for married vs. non-married has also been found in previous studies for general mortality risk (Roelfs et al., 2011). However, also in the elderly population, CHD risk was lowest in the married men and women, in line with a previous meta-analysis of general mortality in the older population (Manzoli et al., 2007). Interestingly, we found that in the older participants, the CHD difference between marital history categories were typically larger in females than in males. This was particularly true towards the end of the study period. Further, the adjustment for socioeconomic indicators slightly widened the CHD differences between the categories of marital history in older women contrasting men. This indicates that the dynamics between partnership and socioeconomic factors in older women is different than in men and in middle-aged women: non-marital groups are socioeconomically more privileged among older women than the married. This may suggest that better educated women in older birth cohorts decided to remain non-married in order to fully participate in employment and develop careers.

Although our results lend some support to the hypothesis that selection to partnership contributes to the associations between partnership status and CHD risk, they do not exclude the possibility that partnership also affects CHD risk. These effects may be mediated by health behaviors as was suggested by the finding that living in an intimate relationship can suppress the genetic susceptibility to heavy alcohol use (Barr et al., 2019) and the effect of decreasing alcohol price on the risk of alcohol related deaths (Herttua et al., 2011). Further, both positive (Alexander et al., 2021) and negative emotions (Arias et al., 2020) have widespread effects on human neurobiology, which can directly affect CHD risk through, for example, hormonal mechanisms, although the biological pathways are still poorly understood. It is well known that depression (Gan et al., 2014) and social isolation (Valtorta et al., 2016) are associated with increased CHD risk, and that they are more common in those living alone than those living with a partner (Frech & Williams, 2007). Furthermore, these protective effects of partnership social support on CHD risk may be stronger in marriage than in cohabitation.

Our data have strengths but also limitations. Our data cover the total Finnish population across three decades. This offers strong statistical power to analyze long-term changes in the association between marital history, using a detailed classification, and CHD incidence. Further, there is no non-participation or drop out in our data, which could otherwise create bias in the results. It is also a major advantage that the Finnish registers allow to identify not only legal marriages, divorces and widowhood but also cohabitation, which has become an important form of partnership in Europe and the USA (Thomson, 2014). However, needing to rely only on register based data is also a weakness since we do not have any direct information on health behaviors or social support from partners. Thus, we can only speculate on possible mediating factors between partnership history and CHD risk. Furthermore, we cannot identify same-sex cohabiting couples and legal registration for same-sex couples become possible in Finland only in 2003 after which year they were recorded as marriages. Thus, those living in same-sex partnerships are incorrectly classified as singles in our data. There is evidence that same-sex marriages offer at least the same level of social and emotional support as different-sex marriages (Thomeer et al., 2021). Thus, we assume that those living with a same-sex partner have the same health benefits as those in different-sex partnerships, and consequently this misclassification probably decreases the CHD incidence in the never married category. Thus, without this misclassification, the differences between never married and those married or cohabiting would probably be larger than we observed in this study.

In conclusion, both current partnership status and marital history are associated with CHD risk. Being married is associated with lower CHD risk exceeding the benefits of cohabitation whereas divorce can have long-lasting negative effects on CHD risk even in those currently living with a partner. These results emphasize that marriages should be better supported in social policy and legislation since marriage generally creates a healthy environment. Divorced and never married men and women may suffer from health problems and should be recognized in a society as a group needing special support. However, because these associations can only partly be explained by adult socioeconomic factors or childhood family background shared by siblings, further research is needed to identify other pertinent factors underlying these associations. Finally, marital status differentials have increased considerably over time, and these differentials should be better recognized as substantial driver of health inequalities in addition to the more commonly investigated socioeconomic disparities.

## Author statement

Karri Silventoinen: Conceptualization, Writing - Original Draft; Kaarina Korhonen: Conceptualization, Formal analysis, Writing - Review & Editing Pekka Martikainen: Conceptualization, Writing - Review & Editing, Resources, Funding acquisition.

## Ethical approval

The data used in the study is based on Finnish registers with data linkage by the Statistics Finland (permission TK-53-1490-18). The data and its use for research have been approved by the Statistics Finland.

## Declaration of competing interest

None to declare.
